# Leveraging mobile phone surveys during the COVID-19 pandemic in Ecuador and Sri Lanka: Methods, timeline and findings

**DOI:** 10.1371/journal.pone.0250171

**Published:** 2021-04-15

**Authors:** Rachael Phadnis, Champika Wickramasinghe, Juan Carlos Zevallos, Stacy Davlin, Vindya Kumarapeli, Veronica Lea, Juliette Lee, Udara Perera, Francisco Xavier Solórzano, Juan Francisco Vásconez

**Affiliations:** 1 Division of Global Health Protection, Center for Global Health, Centers for Disease Control and Prevention, Atlanta, Georgia, United States of America; 2 CDC Foundation, Atlanta, Georgia, United States of America; 3 Non-Communicable Disease Bureau, Ministry of Health, Colombo, Sri Lanka; 4 Ministry of Health, Quito, Ecuador; 5 CDC Foundation—Ministry of Health of Ecuador Project Coordinator, Atlanta, Georgia, United States of America; Iwate Medical University, JAPAN

## Abstract

Effective and rapid decision making during a pandemic requires data not only about infections, but also about human behavior. Mobile phone surveys (MPS) offer the opportunity to collect real-time data on behavior, exposure, knowledge, and perception, as well as care and treatment to inform decision making. The surveys aimed to collect coronavirus disease 2019 (COVID-19) related information in Ecuador and Sri Lanka using mobile phones. In Ecuador, a Knowledge, Attitudes and Practices (KAP) survey was conducted. In Sri Lanka, an evaluation of a novel medicine delivery system was conducted. Using the established mobile network operator channels and technical assistance provided through The Bloomberg Philanthropies Data for Health Initiative (D4H), Ministries of Health fielded a population-based COVID-19-specific MPS using Surveda, the open source data collection tool developed as part of the initiative. A total of 1,185 adults in Ecuador completed the MPS in 14 days. A total of 5,001 adults over the age of 35 in Sri Lanka completed the MPS in 44 days. Both samples were adjusted to the 2019 United Nations Population Estimates to produce population-based estimates by age and sex. The Ecuador COVID-19 MPS found that there was compliance with the mitigation strategies implemented in that country. Overall, 96.5% of Ecuadorians reported wearing a face mask or face covering when leaving home. Overall, 3.8% of Sri Lankans used the service to receive medicines from a government clinic. Among those who used the medicine delivery service in Sri Lanka, 95.8% of those who used a private pharmacy received their medications within one week, and 69.9% of those using a government clinic reported the same. These studies demonstrate that MPS can be conducted quickly and gather essential data. MPS can help monitor the impact of interventions and programs, and rapidly identify what works in mitigating the impact of COVID-19.

## Introduction

Coronavirus disease 2019 (COVID-19) has altered how survey data are currently collected to protect the health and safety of those conducting surveys and stop potential disease transmission [1]. Effective and rapid decision making during all stages of the pandemic require data not only about infections, but also about human behavior [2]. Mobile phone surveys (MPS) can offer the opportunity to collect real time data on behavior, exposure, knowledge and perceptions as well as care, treatment, and resource allocation. Given that mobile phone penetration is high, MPS have become popular in low- and middle-income countries (LMICs) [3–6]. MPS can be conducted without in-person contact with respondents, which makes them particularly suitable during a pandemic [7, 8]. Furthermore, when faced with rapidly moving infectious disease outbreaks, assessing behavior, knowledge and perceptions must be accomplished in a short time frame in order to inform the public health response. Rapid MPS, which demand minimal human resources, could reach large numbers of respondents in a short time frame and is a valuable tool to assess knowledge and perceptions of an infectious disease during an outbreak as well as monitor trends over time [9].

COVID-19 has altered population movement, habits, and practices [10]. Citizens have had to adapt where and how they live, work, eat, and visit [11]. Furthermore, the pandemic has hindered surveillance and data collection efforts globally [10, 12, 13].

Ecuador has been badly affected by COVID-19 [14]. On February 29, 2020, Ecuador confirmed its first imported case of COVID-19 with the return of travelers from Spain [15]. Government officials were proactive in their response and swift action to curb the spread of COVID-19 was adopted. A national health emergency was declared on March 11, 2020. All non-essential activities were suspended indefinitely, the use of virtual platforms was encouraged for academic activities, medical care and jobs, a national curfew was set, and land, air and sea borders were closed to citizens and non-citizens for 21 days [16–19]. Despite these measures, the virus continued spreading across the Ecuadorian provinces. As of late October, Ecuador had reported 162,178 cases with 12,573 deaths [20].

On January 27, 2020, the Ministry of Health and Indigenous Medical Services in Sri Lanka confirmed the first COVID-19 hospitalization in Sri Lanka [21]. The first locally acquired case of COVID-19 was confirmed on March 11, 2020 [22]. In response to the pandemic, the government of Sri Lanka swiftly restricted travel and established 45 quarantine centers across the country. In addition, a series of curfews were imposed beginning in mid-March [23]. In addition to a strictly enforced early lockdown, relatively high testing rates and well-established healthcare and public health surveillance systems helped prevent many cases and deaths [24]. As of late October, Sri Lanka had reported 8,413 cases with 19 deaths [20].

The Bloomberg Philanthropies Data for Health Initiative (D4H) partners with LMIC governments to strengthen their public health data and improve the use of this information to make policy decisions and public health investments. One arm of the initiative explores innovative approaches to non-communicable disease (NCD) surveillance, including the use of MPS to monitor risk factors associated with NCDs [25]. The authors developed an open source survey tool, Surveda, in collaboration with RTI International and Innovative Support to Emergencies Diseases and Disasters (InSTEDD) specifically for this initiative because no existing software met the project needs. Surveda is an open source tool and thus freely available to individuals and organizations interested in conducting their own MPS. Since the launch of the initiative in 2015, seven countries, including both Ecuador and Sri Lanka, have fielded population based MPS using Surveda to monitor major risk factors for premature death. The data collected as part of the initiative is owned by the respective countries. Surveda is a web-based tool that can collect data on many different topics. Surveda can collect data through text messaging (SMS), interactive voice response (IVR) phone calls, and mobile web. In addition, a combination of these modes can be used, commonly referred to as mixed mode data collection. During the COVID-19 pandemic, the Data for Health Initiative was well positioned to assist countries in providing critical COVID-19 data for their pandemic response. Ministries of Health in Ecuador and Sri Lanka used Surveda to quickly deploy COVID-19 related MPS.

The survey in Ecuador allowed the Ministry of Health to use Surveda to study baseline knowledge, beliefs, and practices in relation to COVID-19 containment measures among the adult population aged 18 years and above in Ecuador to determine whether the national health education program for COVID-19 prevention and control was working as intended and assess the impact of mitigation strategies. The survey in Sri Lanka allowed the Ministry of Health to use Surveda to evaluate the use and performance of a medication delivery service during the pandemic among the adult population aged 35 years and above in Sri Lanka to determine if the service was functioning as designed. The medication delivery system was designed to supply NCD medications during the all-island curfew, which required that residents stay indoors for approximately two months in an effort to curb the spread of COVID-19. This study examines the results from these MPS and presents the methods and timelines for these activities.

## Methods

### Survey design

The study protocol and procedures for the MPS were reviewed and approved by the Office of the Associate Director for Science within the Center for Global Health at the Centers for Disease Control and Prevention under the D4H initiative. Approval was received for a Non-research Determination. Using the established mobile network operator (MNO) channels and technical assistance provided through D4H, Ministries of Health in Ecuador and Sri Lanka fielded population based COVID-19-specific MPS using Surveda. Data collection was anonymous, and the surveys were self-administered using the prompts in IVR. Consent was verbal and asked as the first IVR question after language selection. Participants responded by choosing one of two prompts that corresponded with “yes” or “no”. Those who selected “no” were thanked, and the call was ended. Consent was recorded and maintained as part of the final dataset. In Ecuador, the MPS addressed KAPs during the pandemic, availability and barriers to testing and symptomology of current illnesses of adults aged 18 years and older. The Ecuadorian Ministry of Health developed unique questions and utilized questions that were previously validated and used in other recent COVID-19 research [26, 27]. In Sri Lanka, the MPS assessed the use and performance of a medicine delivery system for NCD patients aged 35 years or older. The age eligibility criteria for Sri Lanka was higher given that our target population was those with a need for NCD medicine. The questionnaire topics included the need for prescription medications during the pandemic, usage of the medication delivery system at government clinics and private pharmacies, and the assessment of the delivery system’s speed. These survey questions were developed by the Sri Lankan Ministry of Health specifically for this survey.

The sample designs for both MPS employed a two-phase sampling strategy. In the first phase, a random sample of mobile phone numbers (MPNs) from a frame of active subscribers, excluding those mobile phone numbers that were registered on national Do Not Call registries in Ecuador and Sri Lanka, was provided by a third-party company named Sample Solutions, Inc. In the second phase, respondents from the first phase were stratified into sex strata proportional to the general population. The random digit dial (RDD) generated list of MPNs was uploaded into Surveda and each MPN received a maximum of three contact attempts via IVR, 26 hours apart. These contact attempts included the initial attempt to begin the survey, and any follow-up attempts to begin the survey, as well as re-contacts if the survey was cut-off by the respondent (e.g. the respondent hung up or had poor connectivity). Reverse billing, which prevents respondents from being charged for airtime while responding to the survey, was set up with the MNOs in each country and airtime costs were paid for by the initiative. Individuals who fully completed the survey were provided with a small incentive of $1 USD in Ecuador and no incentive in Sri Lanka. Completed interviews were defined as answering all survey questions. Partial interviews were defined as completing at least one topic area in the Ecuador survey and at least one question in the Sri Lanka survey and not finishing the survey. A summary of the design and data collection procedures for Ecuador and Sri Lanka can be found in Table 1.

**Table 1:** COVID-19 mobile phone survey design in Ecuador and Sri Lanka.

	Ecuador	Sri Lanka
**Mode**	IVR	IVR
**Mobile Network Operators**	CNT, Claro, and Movistar subscribers	Airtel, Dialog, Etisalat, Hutch and Mobitel subscribers
**Strata**	2 strata: male, female	2 strata: male, female
**Questionnaire**	Consisted of 31 questions and was administered in Spanish	Consisted of 24 questions and was administered in Sinhala, Tamil and English
**Contact times**	All 7 days of the week, between 8am and 8pm each day	All 7 days of the week, between 8am and 8pm each day
**Cost to Respondents**	None	None
**Incentives**	$1 USD	None
**Tool and Hosting**	Surveda, with data hosted at the Ecuador Ministry of Health	Surveda, with data hosted at the Sri Lanka Ministry of Health

The Ecuador COVID-19 MPS implementation process consisted of three stages: 1) planning and preparation, 2) pre-test, and 3) full-scale data collection. The planning and preparation phase lasted three weeks. The questionnaire consisted of 31 questions, including consent, and was administered in Spanish. Ecuador’s MPS did not require ethical approval given that the study was considered public health surveillance. Pre-test data collection preceded full-scale data collection. Pre-test included a sample of 1,000 MPNs with proportional allocation to the mobile network market share in Ecuador. The respondent sample size was determined using the standard sample formula: $n=\frac{{\left(1.96\right)}^{2}P(1-P)}{{\left[MOE(\hat{P})\right]}^{2}}*[{Deff}_{o}]$ which takes into consideration the prevalence of the risk factor, precision of the estimate desired margin of error, and design effect. Considering this was a multi-risk survey a prevalence of 50% was assumed in the determination of the sample size. To ensure that precision requirements were met or exceeded for each of the strata and the overall population estimates, the sample size target in each stratum was adjusted by the proportion of the general population using the smallest population stratum as the reference. The total number of required interviews was calculated as 1,036 allocated proportionally to the mobile network market share in Ecuador.

The Sri Lanka COVID-19 MPS was conducted following the same 3-step process. The planning and preparation phase took approximately five weeks. The questionnaire consisted of 24 questions, including language selection and consent. The questionnaire, was administered in Sinhala, Tamil, and English with the respondent selecting their preferred language. The planning phase included ethical review approval from the Ethics Review Committee at Sri Lanka Medical Association. Pre-test data collection, n = 39 respondents, preceded full-scale data collection, and the respondent sample size using the same calculation mentioned above resulted in a sample size calculation of 1,975 interviews allocated proportionally to the mobile network market share in Sri Lanka.

### Implementation timelines

**Fig 1:**
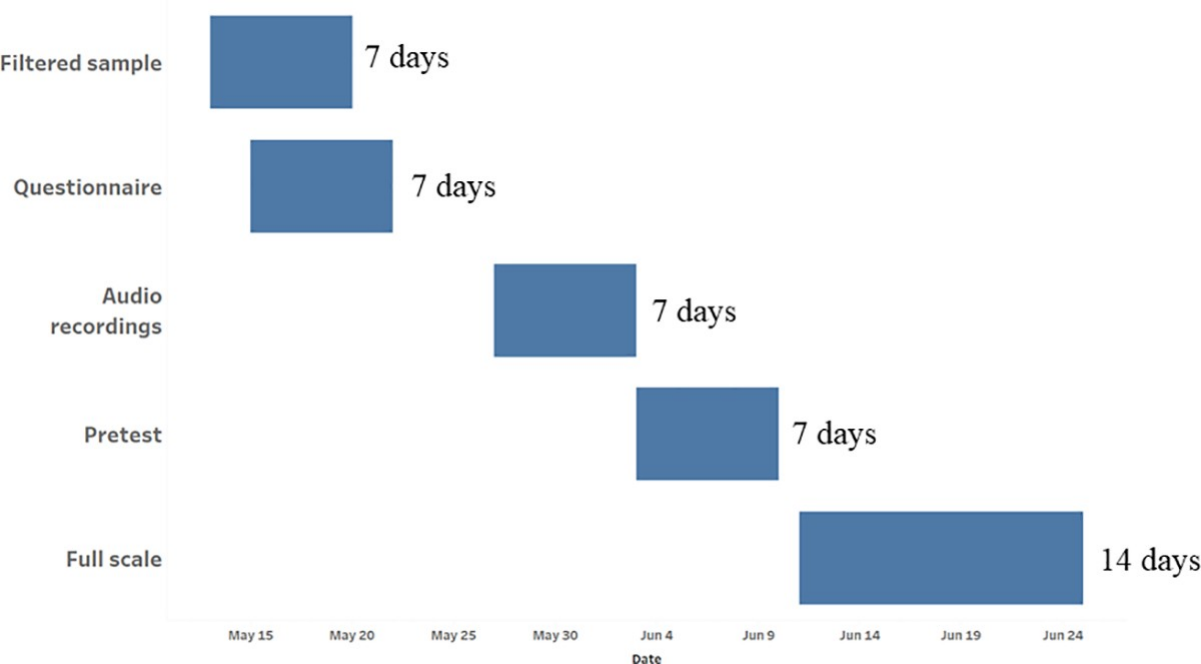
Ecuador planning and data collection timeline.

The Ecuador COVID-19 MPS planning phase consisted of filtering the sample of MPNs to remove non-active numbers, finalizing the 31-item questionnaire and programming the questionnaire in Surveda, which also included securing audio recordings and uploading them into Surveda. Full-scale data collection was completed quickly in 14 days, beginning on June 11^th^ and ending on June 25^th^. Surveda dialed approximately 1,030 MPNs a day and collected an average of 84.6 interviews per day of data collection. Please see Fig 1 for additional details regarding the planning and data collection activities and timeline. The Ecuador COVID-19 MPS used the existing MNO channel used to conduct the NCD MPS in February 2020. The infrastructure and contract with the in-country MNO was maintained at a minimal cost and used to quickly conduct the COVID-19 related MPS. Pre-test data collection was completed prior to full-scale data collection to confirm the functions of the previously established MNO channel as well as test the questionnaire. The pre-test confirmed that scheduled calls were being sent out by Surveda, that the MNO channel could handle the volume of calls being sent out, that mobile phone numbers were tried for the designated number of times and that data were being recorded correctly.

**Fig 2:**
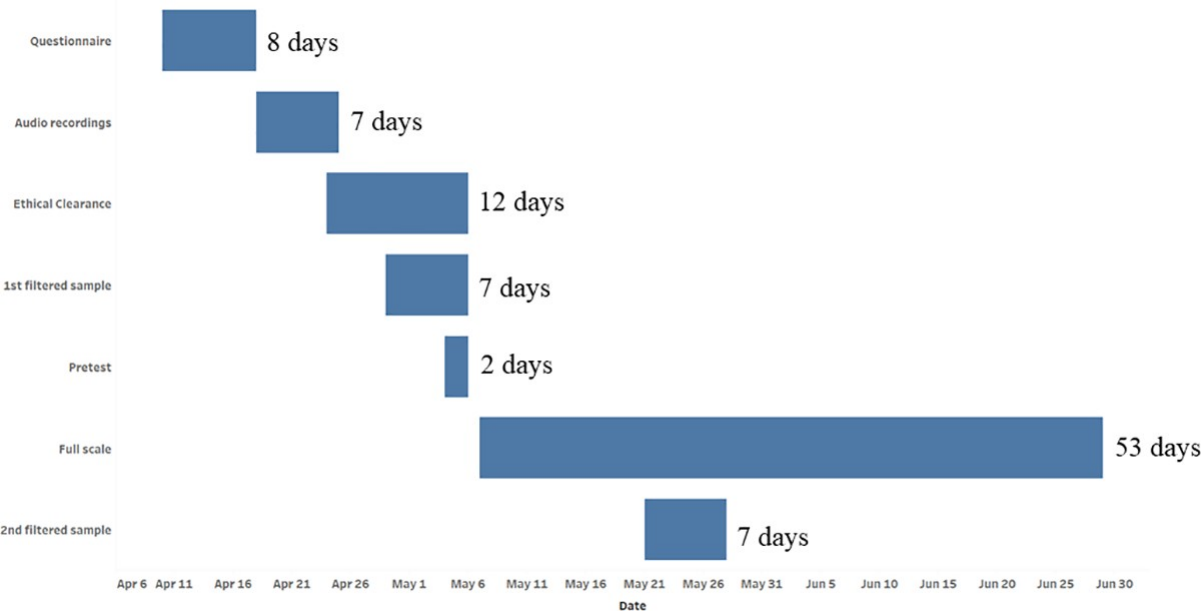
Sri Lanka planning and data collection timeline.

The Sri Lanka COVID-19 MPS planning phase included ethical review approval, filtering the sample of MPNs to remove non-active numbers, and finalizing the 24-item questionnaire. Programming the questionnaire included securing audio recordings in three languages and uploading them into Surveda. Full-scale data collection was completed in 44 days. Please see Fig 2 for additional detail on the planning and data collection timeline. The Sri Lanka COVID-19 MPS used the existing MNO channel used to conduct the NCD MPS from August to October 2019. The infrastructure and contract with the in-country MNO was maintained at a minimal cost in preparation of a repeat NCD MPS, planned for the spring of 2020. The pandemic delayed the repeat NCD MPS and the Sri Lanka Ministry of Health quickly pivoted to use the MNO channel to conduct the COVID-19 MPS to evaluate the newly implemented medicine delivery system. Pre-test data collection preceded full scale data collection, which began on May 7, 2020, and was completed on June 29, 2020. Similarly, the pre-test confirmed that the MNO channel was sending surveys to potential respondents to each of the MNOs, that Surveda was functioning correctly, and data were being recorded correctly. Full scale data collection was paused on May 9, 15–22 and 26–27 to facilitate channel upgrades with the MNO providers. Surveda dialed 380,458 MPNs in 44 days of data collection, averaging approximately 8,646 calls and 113 interviews per day.

### Data analysis

Following data collection, the sample was adjusted to the 2019 United Nations Population Estimates to produce population-based estimates by age and sex. Sample demographics defined weighting classes to ensure that the final weights summed to the specified population totals. These calibrated weights are the final adjusted sample weights that were used for analysis.

In addition, several types of data quality checks were performed, including confirming that the skip logic worked as specified in the questionnaire, checking the amount of time needed to complete each survey, checking that there were no invalid values and assigning final disposition codes, which defined the ultimate outcome of contact attempts for an individual respondent (e.g., whether a respondent was a breakoff, unresponsive, or a partial complete). For binary and categorical response options, the percentage of participants who selected each response was computed using the weighted data. For all proportions, two-sided 95% confidence intervals were calculated. For sex comparisons, we conducted Rao-Scott chi-square tests.

### Response rate calculations

For both COVID-19 MPS, we calculated the following response rates: Phase 1 response rate, Phase 2 response rate, overall response rate, and refusal rate. The Phase 1 response rate was defined as the number of potential respondents screened by interview, both eligible (IELIG) and ineligible (IINEL) divided by the number of randomly selected mobile phone numbers that were dialed, including Unknown MPN status (UH), unknown respondent eligibility and Unknown Refusal (UO). Note that ineligible respondents include respondents less than 18 years of age for Ecuador or 35 years of age for Sri Lanka or respondents age 18 or 35 or greater who matched a stratum where the sample size had been achieved, and who were excluded from participation. The Phase 1 response rate is defined as follows:

$${RR1}_{P1}=\frac{I_{ELIG}+I_{INEL}}{I_{ELIG}+I_{INEL}+UH+UO}$$

The Phase 2 response rate was a modified version of the American Association for Public Opinion Research (AAPOR) RR6 response rate [28]. The Phase 2 response rate was defined as the number of interviews, complete (I) and partial (P), divided by the number of respondents from Phase 1 who are eligible for the interview, including those who provided a complete (I) or partial (P) interview, and those who broke off (NC) after beginning Phase 2. In both COVID-19 MPS, it was impossible to obtain R (eligible refusals/break-offs) and O (eligible other non-interview). As a result, the AAPOR RR6 response rate is reduced to:

$$RR6=\frac{\left(I+P\right)}{(I+P)+NC}$$

## Results

### Response rates

**Fig 3:**
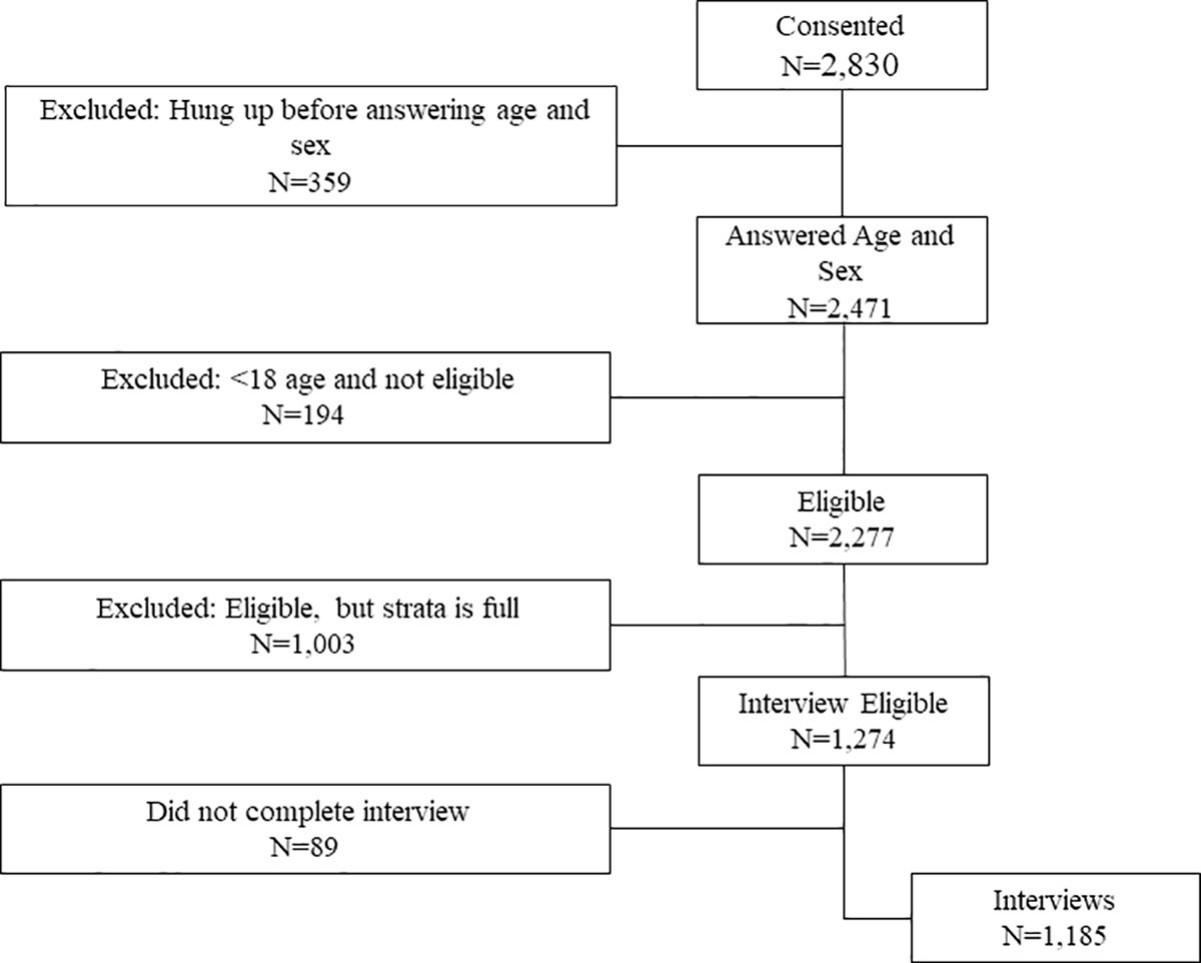
Ecuador COVID-19 mobile phone survey data flow.

In Ecuador, Surveda dialed 14,421 MPNs. Out of these, 2,830 consented to participate and 2,471 provided the age and sex information necessary to be eligible to participate. Of these, 194 were ineligible due to age, and 1,003 respondents of eligible age were rejected due to stratum sample size being full. The result was 1,274 eligible respondents, of which 1,185 provided complete or partial interviews. Fig 3, below, provides additional information on the data flow for the Ecuador COVID-19 MPS.

**Fig 4:**
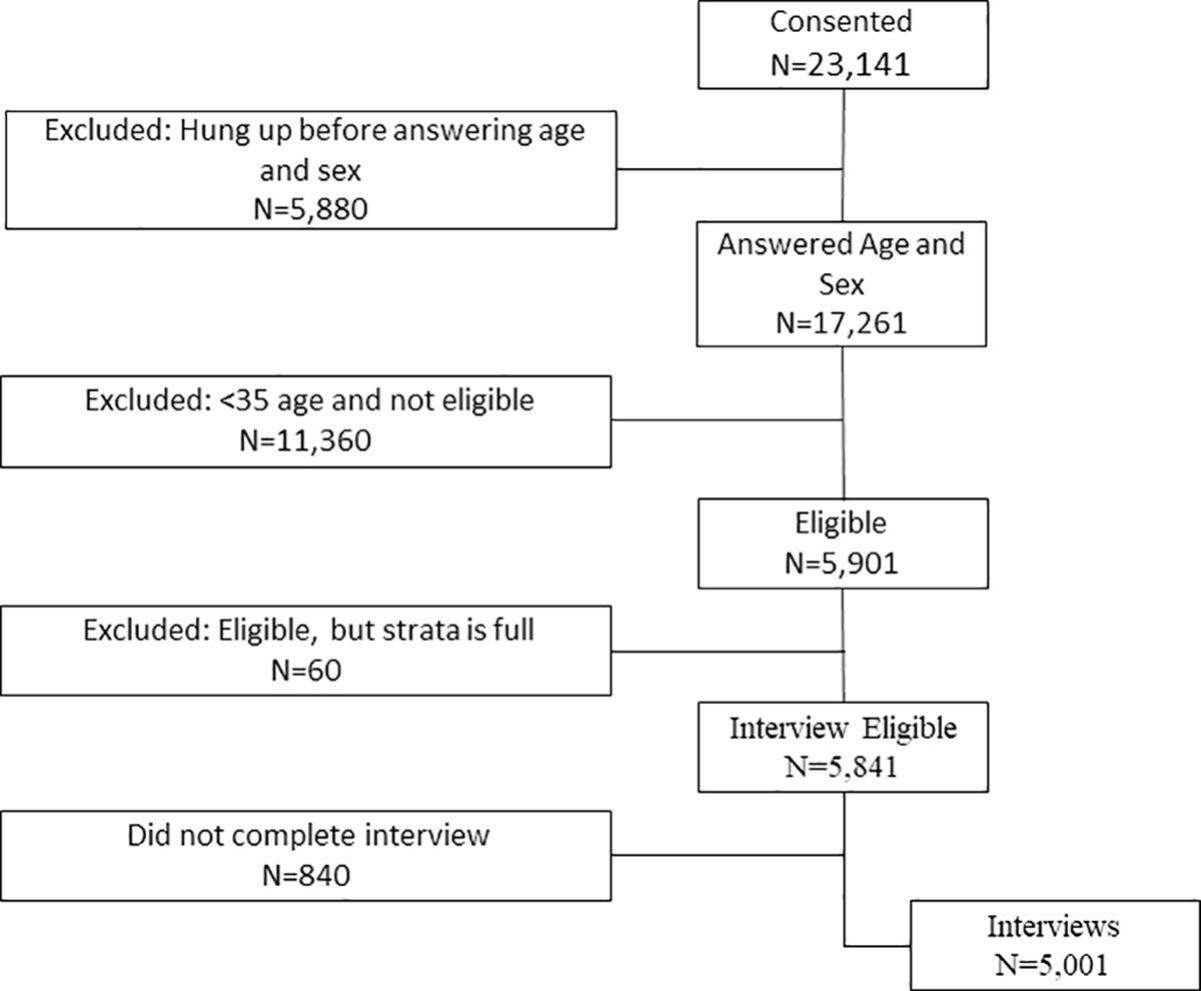
Sri Lanka COVID-19 mobile phone survey data flow.

In Sri Lanka, Surveda dialed 380,458 MPNs. Of these, 23,141 consented and 17,261 provided the age and sex information necessary to be eligible to participate. Of these, 11,360 were ineligible (less than 35 years old), and 60 respondents of eligible age were rejected due to stratum sample size being full. The result was 5,841 eligible respondents, of which 5,001 provided complete or partial interviews. Given the need to evaluate the responses from those who had used the medicine delivery service, the number of interviews collected exceeded the sample size calculation. See Fig 4 for more information on the data flow.

For the Ecuador COVID-19 MPS, the Phase 1 response rate was 17.1%. For the Sri Lanka COVID-19 MPS, the Phase 1 response rate was 4.5%. Table 2 shows the response rates for both countries.

**Table 2:** Response rates.

	Ecuador	Sri Lanka
Phase 1 Response Rate^a^	17.1%	4.5%
Phase 2 Response Rate^b^	93.0%	85.6%
Overall Response Rate^c^	15.9%	3.9%
Refusal Rate^d^	5.1%	2.0%

^a^Number of MPNs screened/Number of MPNs dialed

^b^Modified AAPOR RR6

^c^Product of Phase 1 and Phase 2

^d^AAPOR Refusal Rate #1

The Phase 2 response rate for Ecuador was 93.0%. The Phase 2 response rate for Sri Lanka was 85.6%. We also calculated the overall response rate, which is the product of the Phase 1 and Phase 2 response rates. For the Ecuador MPS, the overall response rate was 15.9%. For the Sri Lanka MPS, the overall response rate was 3.9%. Lastly, we also calculated the Refusal Rate #1 from AAPOR [28]. The refusal rates for Ecuador and Sri Lanka were 5.1% and 2.0% respectively. Table 2 shows the Phase 2 response rate, overall response rate and refusal rate for both countries.

### Respondent characteristics

A total of 1,185 adults aged 18 or older completed the survey in Ecuador. The majority (85.2%) of respondents completed the interview and 14.9% provided a partial interview. A little over half (54.0%) of the sample were female and 46.0% were male. Almost half (45.8%) of the sample were between the ages of 18–29. With respect to the socio-demographic structure, we can say that our sample contains a higher proportion of females and younger adults compared to the country’s population. Table 3 shows the respondent demographics for the Ecuador MPS.

**Table 3:** Respondent demographics: Ecuador COVID-19 MPS.

Sample Characteristics			Population^a^
Number of respondents	1185	** **	16.9 million
**Interviews, n (%)**			
Complete	1009	85.1%	N/A
Partial	176	14.9%	N/A
**Sex, n (%)**			
Female	640	54.0%	51.4%
Male	545	46.0%	48.6%
**Age, n (%)**			
18–29	543	45.8%	31.1%
30–44	419	35.4%	31.0%
45+	223	18.8%	38.0%

^a^Information obtained from 2020 Census

A total of 5,001 adults aged 35 or older completed the survey in Sri Lanka. The majority (94.7%) of respondents completed the interview and only 5.3% provided a partial interview. More than half (65.0%) of the sample were male and 35.0% were female. Approximately half (52.4%) of the sample were between the ages of 35–44. The majority (86.8%) of the interviews were completed in Sinhala. With respect to the socio-demographic structure, our sample contains higher proportions of males and younger adults than the population. Table 4 shows the respondent demographics for the Sri Lanka MPS.

**Table 4:** Respondent demographics: Sri Lanka COVID-19 MPS.

Characteristics			Population^a^
Number of respondents	**5001**	** **	22.9 million
**Interviews, n (%)**			
Complete	4736	94.7%	N/A
Partial	265	5.3%	N/A
**Sex, n (%)**			
Male	3253	65.0%	52%
Female	1748	35.0%	48%
**Age, n (%)**			
35–44	2622	52.4%	43%
45–54	1515	30.3%	31%
55+	864	17.3%	26%

^a^Information obtained from and Sri Lanka Census of Population and Housing 2018 Projections and https://www.cia.gov/library/publications/the-world-factbook/attachments/summaries/CE-summary.pdf

### Ecuador COVID-19 mobile phone survey findings on practices

The Ecuador COVID-19 MPS found that compliance with mitigation strategies was mixed at the time of the survey. Overall, about half (51.8%) of adult Ecuadorians reported they were avoiding public places with no differences by sex. More than half (62.6%) reported avoiding public transportation; males reported similar rates as females, (63.3% and 69.9% respectively). More than three-quarters (79.0%) reported avoiding social contact or practicing physical distancing with no differences by sex. Nine out of ten (90.8%) adult Ecuadorians reported they had left their home in the previous seven days to shop or perform permitted activities. Overall, of those who reported leaving their home, 52.4% left on one or two days, 20.3% left on three or four days and only 18.1% left on five or more days in the previous seven days. Men were statistically significantly more likely than women to have left the home in the last seven days and more frequently, 3–4 days or more than 5 days in the last week. Women were more likely to have either not left home at all or on only 1–2 days per week. Overall, 96.5% of adult Ecuadorians reported that they wore a face mask or face covering when leaving the home with no differences by sex. Table 5 presents key findings on practices overall and by sex.

**Table 5:** Ecuador COVID-19: Survey findings on practices.

	Overall				Males				Females				
	n	%	(95% CI)		n	%	(95% CI)		n	%	(95% CI)		p-value
Avoided public places	1146	51.8	(48.5	, 55.0)	526	50.2	(45.6	, 54.8)	620	53.3	(48.7	, 57.8)	0.3594
Avoided public transportation	1144	62.6	(59.4	, 65.8)	526	63.3	(58.9	, 67.8)	618	61.9	(57.4	, 66.4)	0.6512
Avoided social contact	1130	79.0	(76.2	, 81.7)	515	77.6	(73.6	, 81.5)	615	80.3	(76.5	, 84.0)	0.3353
Left home in the last 7 days	1111	90.8	(88.8	, 92.8)	505	93.9	(91.6	, 96.2)	606	87.9	(84.7	, 91.1)	**0.0025**
Left home 1–2 days	1111	52.4	(49.1	, 55.7)	505	48.7	(43.9	, 53.4)	606	56.0	(51.4	, 60.6)	**0.0297**
Left home 3–4 days	1111	20.3	(17.6	, 23.0)	505	23.1	(19.1	, 27.1)	606	17.7	(14.2	, 21.2)	**0.0479**
Left home ≥ 5 days	1111	18.1	(15.6	, 20.6)	505	22.2	(18.3	, 26.0)	606	14.2	(11.0	, 17.4)	**0.0018**
Left home 0 days	1111	9.2	(7.2	,11.2)	505	6.1	(3.4	, 8.4)	606	12.1	(8.9	, 15.3)	**0.0025**
Wore face mask/covering when leaving the home	1004	96.5	(95.3	, 97.7)	470	96.1	(94.3	, 98.0)	534	96.9	(95.4	, 98.3)	0.545

### Sri Lanka COVID-19 mobile phone survey findings

The Sri Lanka COVID-19 MPS evaluated the newly implemented medicine delivery system that was put in place to supply NCD medications during the all-island curfew. Approximately 24% of Sri Lankans over the age of 35 needed to obtain medicine for NCDs during the curfew. About 18% of Sri Lankans knew about the medicine delivery service. Of those who knew about the service, 3.8% used the service to receive medicines from a government clinic, 12.6% used the service to receive medicines from a private pharmacy and 2.0% received medicines from both. Nearly ninety-sex percent (95.8%) of those who used a private pharmacy received their medicine within one week and 69.9% of those using a government clinic reported the same. The majority of Sri Lankas who used a private pharmacy thought that the bill was reasonable. There were no differences by sex for all indicators except among those who received their medication from both private and public pharmacies (males 1.2% vs. 2.6% females) and thought costs for medication was reasonable (males 90.4% vs. 96.0% females respectively). Table 6 presents key findings overall and by sex.

**Table 6:** Sri Lanka COVID-19: Survey findings.

	Overall				Males				Females				
	n	%	(95% CI)		n	%	(95% CI)		n	%	(95% CI)		p-value
Needed medicine	5001	24.0	(22.3	25.8)	3253	22.3	(20.6	24.1)	1748	25.5	(22.7	28.4)	0.0637
Knew about the medicine delivery service	4861	18.1	(16.5	19.7)	3153	17.0	(15.3	18.6)	1708	19.0	(16.4	21.6)	0.2090
Used the medicine delivery service from a private pharmacy	4669	12.6	(11.1	14.0)	3040	11.2	(9.8	12.6)	1629	13.8	(11.4	16.2)	0.0729
Used the medicine delivery service from a government clinic	4696	3.8	(3.0	4.6)	3058	3.3	(2.5	4.0)	1638	4.3	(2.9	5.7)	0.2052
Used the medicine delivery service from a private pharmacy and a government clinic	4700	2.0	(1.3	2.6)	3061	1.2	(0.8	1.7)	1639	2.6	(1.4	3.7)	**0.0337**
Received medicine from a private pharmacy within 1 week	361	95.8	(92.4	99.3)	223	98.2	(96.3	100.0)	138	94.1	(88.3	99.9)	0.1835
Received medicine from a government clinic within 1 week	69	69.9	(55.0	84.9)	40	67.3	(50.5	84.0)	29	71.2	(50.4	92.1)	0.7691
Thought the private pharmacy bill was reasonable	356	93.7	(91.4	96.0)	217	90.4	(86.0	94.9)	139	96.0	(93.7	98.4)	**0.0294**
Received all of their medicines from a private pharmacy	354	77.2	(71.1	83.2)	217	79.4	(73.1	85.8)	137	75.5	(66.1	84.9)	0.4945

## Discussion

The recent proliferation of mobile phone networks and mobile telephone affordability has contributed to the high penetration of mobile phone users and opened new possibilities for data collection. Based on a combination of baseline data from a traditional household survey and subsequent interviews of selected respondents using mobile phones, a growing number of initiatives are now using mobile phone technology to facilitate rapid data collection [5]. Due to the COVID-19 pandemic, many routine surveillance and data collection efforts have been delayed or postponed indefinitely, since traditional methods of primary data collection that rely on face-to-face household interviews have become too risky [13]. However, the Ministries of Health in Ecuador and the Ministry of Health and Indigenous Medical Services Government of Sri Lanka pivoted successfully to utilize existing surveillance infrastructure to conduct COVID-19 related data collection during the pandemic. Rapid data collection, such as MPS, would help facilitate effective and quick decision making during all stages of the pandemic. It was possible to assess knowledge and perceptions of COVID-19 among the general public in Ecuador and to conduct an evaluation of a novel medicine delivery system in Sri Lanka in a short time frame and with minimal human resources. Data collection in Ecuador required 14 days to collect 1,185 interviews throughout the country. In Sri Lanka, data collection took 44 days to collect 5,001 interviews. Data collection in Sri Lanka required more time due to the narrow age target and the population who needed and used the delivery medicine service. During data collection in Sri Lanka, the Surveda dashboard real-time output alerted the data collection team of MNO channel issues, resulting in short pauses to the data collection effort. In terms of data quality, response rates and item non-response rates were similar to the NCD MPS in their respective countries. For example, the Ecuador NCD MPS had an overall response rate of 9.9% and the Sri Lanka NCD MPS conducted in October 2019 had an overall response rate of 7.6%. There was no indication that participants randomly selected responses as quickly as possible to earn the airtime incentive; only one participant took less than 2 minutes to complete the Ecuador COVID-19 questionnaire. In Sri Lanka there was no incentive provided. Response rates for Ecuador were higher than in Sri Lanka, (17.1% and 3.9%, respectively). This may be attributed to the use of nominal incentives in Ecuador which have been shown to increase MPS participation [29]. Our experience in Ecuador and Sri Lanka showed that it is feasible to quickly and cheaply collect good quality data via mobile phones during the COVID-19 pandemic crisis. The strength of these studies lies in the large sample recruited during the COVID-19 pandemic.

Our Phase 2 response rate is a modified AAPOR RR6, which is also referred to as completion rate [28]. When we compare our Phase 2 response rates, 93.0% for Ecuador and 85.6% for Sri Lanka, we find our rates were higher than completion rates from other IVR surveys. Other studies have reported completion rates of 23–75% in IVR surveys [6]. We speculate that given the timeliness of this data collection, respondents were interested in providing information about the COVID-19 pandemic.

The COVID-19 pandemic has led to a massive global public health campaign to introduce mitigation strategies to slow the spread of the virus by encouraging significant shifts in behavior, including avoiding crowded places, practicing physical distancing, and wearing masks in public. In this study, a majority of Ecuadorians reported adhering to most preventive measures as instructed by their national health care authority. This is the first cross-sectional MPS to examine the KAP towards COVID-19 among the general Ecuadorian population. The findings from the Ecuador COVID-19 MPS are useful for policymakers to quickly understand whether the national health education program for COVID-19 prevention and control is working as intended. The data gathered in Ecuador highlighted adherence to mitigation strategies for prevention and control and contributes to the limited data on KAPs in Latin America during the pandemic [30].

In addition, MPS can provide data that can guide government and public health authorities in determining the best course of action to control the COVID-19 pandemic as it progresses. For example, in Ecuador, the MOH used the results of its COVID-19 MPS to strengthen response policies and strategies and guide internal decisions on pandemic response. For example, the MOH reinforced practices that were included in the survey such as use of face masks, reduce exposure in public transportation and public places, and increase free access to COVID-19 testing. In a country where COVID-19 has been compounded by an economic crisis and political instability, the COVID-19 MPS survey results helped reinforce the MOH pandemic policy response and have provided useful inputs for their new communication campaign. Furthermore, in Sri Lanka, COVID-19 MPS survey results were provided to the Pharmaceutical Society of Sri Lanka, and they were advised by the MOH to take corrective action to better deliver medications to the Sri Lankan population. In addition, the results were published on the Ministry of Health and Indigenous Medical Services Government of Sri Lanka Facebook page, where government officers and the public would be able to provide comments. The Deputy Director General of the NCD Unit, Dr. Wickramasinghe, noted, “We never get real-time feedback, so our decisions are based on what some people have told us or complained about. This time, we had the opportunity to get population feedback on the medication delivery service. As I oversaw organizing online purchasing of medicine from private pharmacies, this was very useful to me. The results showed that many people have ordered and received medicine through government officers. This is one area I have to seriously plan if the need arises with a second wave or if there is another epidemic. I will be writing a report with a future plan and recommendations and submit to Director General of Health Services and Secretary of Health.” (S. Wickramasinghe, personal communication, August 10, 2020). The availability of real time evaluation data on the medicine delivery service in Sri Lanka was very valuable and will help inform future efforts.

Future MPS applications to assess KAPs and gather other critical information at a national level can help fill the gaps for many countries with high mobile phone penetration [4]. Such surveys can provide governments with data to make informed decisions during the COVID-19 pandemic. MPS provides a promising method to assess and track knowledge and perceptions during a rapidly evolving infectious disease outbreak. Such assessments are crucial to ensure that the public is well informed about COVID-19 and can help to reduce disease transmission, ultimately saving lives. MNOs play a vital role in combatting the pandemic and provide a crucial component of social responsibility that can support governments and the research community during this crisis.

## Limitations

The results of the COVID-19 MPS are based on responses from mobile phone owners rather than the general population. This does result in some selection bias that may be targeting those participants who have more education or higher socioeconomic status. However, mobile phone ownership is high in both countries surveyed- there are 91.25 subscriptions for every 100 people in Ecuador and 115.06 subscriptions for every 100 people in Sri Lanka [31]. In addition, data were based on self-report, and participants may be less likely to report information that is deemed socially or culturally undesirable, resulting in overestimation of adherence to mitigation strategies. However, it should be noted that given the anonymous nature of the data collection, the risk of social desirability bias is reduced. Several MPS challenges such as representativeness, response rates, and increasing survey completeness, should be considered and are discussed elsewhere [6]. Lastly, consistent with other mobile phone surveys, we found higher attrition rates for older respondents aged 45 and older. Continued use of mobile phone surveys in LMICs can strengthen partnerships between MNOs, researchers, and governments and provide valuable data for both routine and novel public health surveillance.

## Conclusion

The widespread extent of mobile phone ownership and subscription rates in LMICs provide new ways to collect data during a pandemic. Mobile phone surveys can be used as a quick and inexpensive method to provide data to support a country’s response during the COVID-19 pandemic. MPS quickly provides continual data to allow for effective and rapid decision making during all stages of the pandemic and complements surveillance efforts done with other tools. MPS can help monitor the impact of interventions and programs and help to rapidly identify what works in mitigating the impact of COVID-19. Future research using MPS over the course of the COVID-19 pandemic could include comparisons of data obtained via MPS to that of face-to-face interviews as well as inclusion of panel surveys, where appropriate. Investigating strategies to increase response rates could also be considered.
